# Menstrual health among adolescents and young adults in rural Haiti

**DOI:** 10.1186/s12978-022-01533-4

**Published:** 2022-12-20

**Authors:** Emily R. Rupe, Jonathan Rodean, Emily A. Hurley, Melissa K. Miller, Marie Daphnee Boncoeur, Abbey R. Masonbrink

**Affiliations:** 1 University of Kansas School of Medicine, 1010 N Kansas St., Wichita, KS 67214 USA; 2 Children’s Hospital Association, Lenexa, KS USA; 3 Department of Pediatrics, Children’s Mercy Hospital, Kansas City, MO USA; 4 University of Missouri-Kansas City School of Medicine, Kansas City, MO USA; 5 Maison de Naissance, Larnage, Haiti; 6 Global Birthing Home Foundation, Leawood, KS USA

## Abstract

**Background:**

Adolescent and young adult (AYA) females in low- and middle-income countries often face disparities in menstrual health (MH). Poor MH and lack of sexual and reproductive health education leads to school absenteeism, increasing risk for adverse psychosocial and educational outcomes. Further, disasters (e.g., earthquakes) are linked with unsafe living environments and sanitation facilities for women. We sought to describe MH perspectives and practices among AYAs in rural Haiti.

**Methods:**

We conducted a cross-sectional survey in two rural communities in Haiti. AYA females aged 14–24 years completed questions on demographics, the Menstrual Practice Needs Scale (36 items; MPNS-36) and the Menstrual Practices Questionnaire (4 items). We performed descriptive statistics and Chi square or Fisher’s Exact tests to compare responses among sub-groups.

**Results:**

Among 200 respondents, the median age was 20 years (IQR 17–22). 51% (95% CI 44%, 58%; 102/200) were currently attending school at least 3 days/week and 97% (94%, 99%; 193/200) were not married. According to the MPNS-36, 68% (62%, 74%; 136/200) of participants had unmet MH needs. Seventy-one (77%) reused some of their menstrual materials during their last menstruation. During their last menstruation, 44% (37%, 50%; 87/200) reported they often or always skipped school because they had their menses, and 31% (25%, 37%; 62/200) sometimes skipped. Many felt always or often worried that someone or something would harm them while they were changing their menstrual materials at home and at school.

**Conclusions:**

Among AYAs in rural Haiti, three-quarters reported menses-related school absenteeism and two-thirds had unmet MH needs. AYA females often lacked a safe environment to change their menstrual materials. Given recent disasters in Haiti, (August 2021 earthquake), safe environments for MH are critically needed to offset risk for poor psychosocial and health outcomes. Future efforts to improve MH among AYAs in Haiti are needed to ensure access to MH resources and school attendance.

**Supplementary Information:**

The online version contains supplementary material available at 10.1186/s12978-022-01533-4.

## Plain language summary

Adolescents and young adult (AYA) females in low- and middle-income countries can often lack adequate access to materials to manage their menstruation, in addition to safe spaces to change those materials. This lack of access, in addition to low levels of reproductive health education, can cause AYAs to miss school, leading to increased risk of worse psychosocial and educational outcomes. In addition, disasters (e.g., earthquakes) are linked with unsafe living environments and sanitation facilities for women. We sought to describe AYA menstrual practices and perspectives on menstrual hygiene in rural Haiti. In two rural communities in Haiti, AYA females aged 14–24 years answered questions on their demographics and menstrual hygiene practices and environments. Two-thirds of AYAs had unmet menstrual hygiene needs and three-quarters reported they skipped school (with any frequency) due to their menses. Further, more than half worried that something or someone would harm them while they were changing their menstrual materials at home and at school. AYA females often lacked a safe environment to change their menstrual materials. Given recent disasters and political unrest in Haiti, (August 2021 earthquake), it is important to improve menstrual hygiene in these LMICs to ensure safe enviornments for managing menstruation and school attendance and ultimately improve psychosocial and health outcomes.

**Supplementary Information:**

The online version contains supplementary material available at 10.1186/s12978-022-01533-4.

## Background

An important component of menstrual health (MH) is access to adequate materials (e.g., sanitary pads, absorbent materials), a safe and private space to change materials, as well as soap and water [1]. However, a majority of adolescent and young adult (AYAs) females in low- and middle-income countries (LMICs) lack access to one or more of these key MH resources [2]. The United Nations Sustainable Development Goals for 2030 highlight MH education and resources for young women as a key component of improving access to water, sanitation, and hygiene (WASH) resources [3, 4]. Further understanding of MH practices and barriers can inform efforts to improve the health and educational outcomes in this population. [4]

Menstrual absorbent preferences among women in LMICs are driven by a number of factors, including access, cost, and convenience [5]. Some women preferentially use disposable sanitary pads due to comfort and lack of odor and concerns about washing and drying reusable absorbents in public spaces [2, 6–8]. However, there are limitations to the use of disposable menstrual materials (e.g., pads, tampons), such as concerns about sanitary disposal and environmental waste. Additionally, access to sanitary pads is often disproportionately concentrated in urban settings and among those with a higher socio-economic status (SES) [2, 9, 10]. When sanitary pads are used, their limited availability, combined with the lack of time and privacy for pad changes, results in prolonged wear, irritation, and chafing [11]. AYAs who do not have access to sanitary pads typically use natural or household material alternatives, such as rags and grass, which are prone to leak, cause soreness, and are perceived as harmful (i.e., concern about infection) among users [11]. In southern Nigeria, for example, adolescent girls reported primarily using sanitary pads (64%) and toilet tissue/paper (22%), with less than 15% using clothes, tampons, or multiple materials as a menstrual absorbent [6]. Additionally, some schools in LMICs may lack private, safe latrines as well as water and soap, which deters girls from changing their absorbents while at school [12]. Poor MH is linked with increased risk for vaginal irritation, discomfort, and urogenital symptoms (even if not confirmed infections), which can negatively impact daily functioning [13]. Further, some menstrual practices may be associated with increased risk for urogenital infection, while higher socio-economic status to support household MH resources may be protective against menstrual-related vaginal infections. [13–15]

Inadequate MH resources are further complicated by poor access to sexual and reproductive health (SRH) education and services, as well as a general lack of parental and school support for SRH [11, 16, 17]. Inadequate MH has also been linked with a decline in school attendance among AYA populations living in LMICs, potentially resulting in lower educational attainment and financial opportunities [18–23]. In rural Kenya, girls reported ‘others’ rather than themselves missed school due to their menstrual symptoms or lack of sanitary menstrual absorbents. Girls also described difficulties concentrating in class due to fear of menstrual odor and leakage and subsequent teasing [11]. However, there is conflicting data on the impact of MH interventions to improve school attendance and related psychosocial outcomes among young women in LMICs [16, 24, 25]. Thus, the effects of poor MH on education among young women are likely more complex than just school absenteeism, and may include higher baseline stress levels, decreased concentration in school, and/or personal health decline [12]. Additionally, disparities in WASH resources, including unsafe sanitation facilities, can lead to an increased risk of gender-based violence (GBV) while girls and women are performing typical hygiene related tasks [26–28]. Unsafe facilities are often dimly lit, isolated, and/or without locks [13–15]. The United Nations defines a disaster as a “serious disruption of the functioning of a community or a society involving widespread human, material, economic or environmental losses and impacts, which exceeds the ability of the affected community or society to cope using its own resources” [29, 30]. Natural and other disasters can further exacerbate safety concerns and GBV among women due to displacement of residents to camps with limited or no access to sanitation facilities [26–28].

Specifically in Haiti where disasters have recently occurred (e.g., August 2021 earthquake), there is little research describing the context of MH among AYAs. Further, Haiti’s waste management and disposal systems often fall short of WASH standards [31]. For example, many families do not have access to private, covered latrines. Adult women in Haiti report concerns about sanitation, hygiene and SRH, including family planning and vaginal infections [32]. Haitian women frequently reported lack of feminine hygiene products as a top reason for school absenteeism (21%) and 97% agreed or strongly agreed there is a need for MH programs in Haiti [33]. To our knowledge, no studies have described MH practices in younger women in in rural Haiti. Our objective in this study was to describe MH practices and identify unmet needs among AYAs living in rural Haiti.

## Methods

### Study design

We conducted a cross-sectional survey of female AYAs in rural Haiti from August 2021 to March 2022. Inclusion criteria included biological females aged 15 to 24 years old who lived in one of two rural communities located in Southern Haiti (near Les Cayes) or Plateau Central (near Des Chappelles) and were capable of providing informed assent/consent. The study was approved by the institutional review board at the affiliated academic institution in the United States and by a local university or community representative according to local procedures in Haiti. All study materials were created in English and translated into Haitian Creole by a bilingual university-level interpreter. Translated documents were reviewed by the local research team and the university faculty representative in Haiti to ensure accuracy and comprehensability. All study procedures were conducted in Haitian Creole.

### Data collection

The research team recruited a convenience sample of AYAs by working with a local community member to identify homes in the community where potential participants may reside. Eligible AYAs were approached at their homes and underwent verbal assent (15–17 years old) or consent (18–24 years old) for study enrollment. Parental consent was waived; however if a parent or guardian was present, they were given brief study information and then asked to provide a private space for assent or consent and study procedures. Similar to past studies, to overcome concerns about limited literacy, the 30-min survey was administered verbally by the study team. Study data were reviewed for completeness and accuracy and entered electronically using Research Electronic Data Capture (REDCap) within one week of data collection by the team member who administered the survey. No participant identifiers were collected. Any paper study materials were destroyed upon study completion. Following survey completion, all participants were given a menstrual hygiene kit and verbal education on kit use.

### Measures

We assessed socio-demographic information with items adapted from a previous SRH study of adolescents in Haiti [34]. These multiple choice questions were used to assess age, marital status, school attendance and home environment (i.e., “What best describes where you live?”). Please see Additional file 1 for the complete survey instrument. We used the “Menstrual Practice Needs Scale (MPNS-36),” a well-validated assessment of menstrual hygiene practices and environments based on responses to 4-point Likert Scale items (Options: Never, Sometimes, Often, Always) [35]. The MPNS-36 contains 36 items that assess (1) material and home environment needs (2) transport and school environment needs (3) material reliability concerns, (4) change and disposal insecurity, (5) reuse needs and (6) reuse insecurity. To assess current MH practices, additional multiple-choice items (n = 4) from the “Menstrual Practices Questionnaire (MPQ)” which were answered by a subset of the study population [35]. These questions were added after data collection was completed at site 1, thus were answered by participants at site 2. To assess MH related school absenteeism, we added a question based on past MH studies that was not in the validated MPNS-36 tool (“During your last menstrual period, how often did you.. I skipped school because I had my menses. Response options: Never, Sometimes, Often, Always”) [20, 23]. Although previously validated in 2020 by a study in Uganda of 538 menstruating adolescent girls, the survey was pre-tested with three AYAs representatives and we provided minor revisions based on feedback to improve clarity [35].

### Statistical analysis

We used descriptive statistics to report proportions of responses to multiple choice and Likert scale questions. All statistical analyses were conducted using SPSS, Version 20 or SAS software v 9.4 (SAS Institute, Cary, NC, USA). Chi square or Fisher’s Exact tests (i.e., when 25% of the expected counts were < 5) were used to compare between participants living in a tent vs house. Because this was an exploratory study with a convenience sample, we did not have a predetermined sample size. We did, however, determine the sample size we achieved would be sufficient to run comparisons between participants living in a tent vs home. Assuming the observed sample size of 178 living in homes and 22 in tents, and assuming a reference proportion of 0.197 responding that they were often or always worried something would harm them when changing their menstrual materials [35], the difference in proportions required to achieve 80% power is a difference of 0.25. The overall and topic MPNS-36 score was calcuated based on previously described methods [35]. The percentage of participants who reported a score of less than 3 was calculated and reported as the percentage of participants with unmet MH needs. To better understand the socio-demographic characteteristics of participants with unmet MH needs, we divided participants into those with low scores and those with high scores and compared the characteristics of the two groups using a chi-square test for association or Fisher exact test (for small sample sizes). There were 26 responses with 3 or more items missing. Per MPNS-36 guidelines, these responses were not scored as either high scoring or low scoring [35], leaving us with a final comparison set of 174 responses. We defined “low scores” as those whose total mean score was at least 1 SD or more below the group mean (i.e., a mean total score < 1.82).

## Results

Among 200 respondents, 102 (51%) reported regular school attendance, 70 (35%) had completed 7–9th grade in primary school and 83 (42%) had completed 3–4th grade in secondary school (10th–11th grade equivalent) (Table 1). The overall mean MPNS-36 score was 2.2 (SD: 0.33); 68% of participants had a mean MPNS-36 score of 3 or less, indicating unmet MH needs. Participants who responded that their religion was “None” were more likely to have a low score (p =  < 0.01) (Additional file 2).

**Table 1:** Participant demographics

	N = 200, n (%)
Age category (years)	
14–17	53 (27)
18–21	76 (38)
22–24	71 (36)
Highest education level (grade)	
1–6th primary school	35 (18)
7–9th primary school	70 (35)
3–4th secondary school	83 (42)
Graduated secondary school	12 (6)
Marital status	
Married	7 (4)
Regular school attendance (attending school at least 3 days a week)	
Yes	102 (51)
Living situation	
House	178 (89)
Tent	22 (11)
Religion	
Catholic	63 (32)
Other Christian religion	96 (48)
Other	1 (1)
None	39 (20)
Did not answer	1 (1)
Sexual orientation	
Gay	0 (0)
Lesbian	0 (0)
Straight/not gay or lesbian	199 (100)
Bisexual	1 (1)
Did not seek care in past year when felt they should	100 (50)

### Material and home environment needs

The mean score for materials and home environment was 2.3 (SD: 0.16) and 50% of participants had unmet needs related to materials and home environment. During their last menstrual period, 39 (20%) reported their menstrual materials were never or sometimes comfortable (Table 2).

**Table 2:** Material and home environment needs (n = 200)

During your last menstrual period how often did you feel that…	Participant responses, n (%)			
	Never	Sometimes	Often	Always
My menstrual materials were comfortable	23 (12)	16 (8)	47 (24)	114 (57)
I had enough of my menstrual materials to change them as often as I wanted to	11 (6)	20 (10)	54 (27)	115 (58)
I was satisfied with the cleanliness of my menstrual materials	9 (5)	5 (3)	58 (29)	127 (64)
I could get more of my menstrual materials when I needed to	16 (8)	29 (15)	61 (31)	93 (47)
I felt comfortable storing my menstrual materials until my next period	24 (12)	24 (12)	75 (38)	76 (38)
I was able to wash my hands when I wanted to	13 (7)	45 (23)	71 (36)	70 (35)
I was able to immediately throw away my used menstrual materials or find a place to store them if they are reusable	2 (1)	33 (17)	73 (37)	91 (46)
I was able to dispose of my used materials in the way that I wanted to	10 (5)	42 (21)	27 (14)	118 (60)
When at home, I was able to change my menstrual materials when I wanted to	9 (5)	4 (2)	86 (43)	100 (50)
When at home, I was satisfied with the place I used to change my menstrual materials	9 (5)	8 (4)	86 (43)	96 (48)
When at home, I had a clean place to change my menstrual materials	7 (4)	2 (1)	88 (44)	102 (51)

All data obtained from both site 1 and 2

Among a subset of the sample who responded to the additional MPQ questions (Site 2, n = 92), a large majority reported use of a disposable sanitary pad (n = 94; 92%) and a cloth or towel (n = 69; 68%) to manage their menses while at home. When away from home, most reported use of a disposable sanitary pad (n = 79; 78%) and a cloth or towel (n = 62; 62%). Among cloth users, half of respondents (n = 45; 50%) used cloths that were bought for the purpose of menstruation, while half (n = 45; 50%) used cloths that were used for something else first. Seventy-one (77%) reused at least some of their menstrual materials during their last menstrual period (data not shown in table).

### Transport and school environment needs

The mean score for transport and school environment needs was 2 (SD: 0.24); 65% of participants had unmet needs related to transport and school environment needs. During their last menstrual period, 87 (44%) reported they skipped school often or always because they had their menses, while 62 (31%) reported they sometimes skipped school (Table 3). Among those who reported regular school attendance (at least 3 days a week, n = 102), 39% often or always reported skipping school due to their menses, while 30% reported sometimes. When at school, 83 (42%) reported they never or sometimes had a clean place to change their menstrual materials.

**Table 3:** Transport and school environment needs (n = 200)

During your last menstrual period how often did you feel that…	Participant responses (n, %)			
	Never	Sometimes	Often	Always
I felt comfortable carrying spare menstrual materials with me outside my home	16 (8)	21 (11)	66 (33)	96 (48)
I felt comfortable carrying menstrual materials to the place where I changed them	17 (9)	15 (8)	76 (38)	91 (46)
When at school, I was able to change my menstrual materials when I wanted to	23 (12)	39 (20)	78 (39)	59 (30)
When at school, I was satisfied with the place I used to change my menstrual materials	24 (12)	52 (26)	69 (35)	54 (27)
When at school, I had a clean place to change my menstrual materials	25 (13)	58 (29)	66 (33)	50 (25)
I skipped school because I had my menses*	50 (25)	62 (31)	61 (31)	26 (13)

All data obtained from both site 1 and 2

*Item not in Menstrual Practice Needs Scale (MPNS)

### Menstrual reliability concerns

The mean score for menstrual reliability concerns was 1.8 (SD: 0.17); 90% of participants had unmet needs related to menstrual material and reliability concerns. Sixty-two respondents (31%) reported they often or always worried their menstrual materials would leak or move around (Table 4).

**Table 4:** Menstrual reliability concerns (n = 200)

During your last menstrual period how often did you feel that…	Participant responses, n (%)			
	Never	Sometimes	Often	Always
I worried that my menstrual materials would allow blood to pass through to my outer garments	39 (20)	98 (49)	38 (19)	24 (12)
I worried that my menstrual materials would move from place while I was wearing them	32 (16)	104 (53)	38 (19)	24 (12)
I worried about how I would get more of my menstrual material if I ran out	57 (29)	105 (53)	25 (13)	12 (6)

All data obtained from both site 1 and 2

### Change and disposal insecurity

The mean score for change and disposal security was 2.2 (SD: 0.17); 92% of participants reported unmet needs related to change and disposal security. More than half of respondents (55%) reported they worried that someone would harm them while they were changing their menstrual materials when at home (Table 5). Participants living in a tent were more likely to report they were often or always worried (n = 22; 46%) something would harm them when changing their menstrual materials at home, compared to those living in a house (n = 178; 21%; p = 0.016).

**Table 5:** Change and disposal insecurity (n = 200)

During your last menstrual period how often did you feel that…	Participant responses, n (%)			
	Never	Sometimes	Often	Always
I worried about where to dispose of my used menstrual materials	136 (68)	42 (21)	12 (6)	9 (5)
I was concerned that others would see my used menstrual materials in the place I disposed of them	80 (40)	81 (41)	18 (9)	19 (10)
When at home, I worried that I would not be able to change my menstrual materials when I needed to	95 (48)	79 (40)	11 (6)	14 (7)
When at home, I worried that someone would see me while I was changing my menstrual materials	71 (36)	88 (44)	24 (12)	16 (8)
When at home, I worried that someone would harm me while I was changing my menstrual materials	89 (45)	66 (33)	26 (13)	18 (9)
When at home, I worried that something else would harm me while I was changing my menstrual materials (eg, animals, insects, unsafe structure)	71 (36)	81 (41)	12 (6)	35 (18)
When at school, I worried that I would not be able to change my menstrual materials when I needed to	64 (32)	101 (51)	23 (12)	11 (6)
When at school, I worried that someone would see me while I was changing my menstrual materials	60 (30)	96 (48)	30 (15)	13 (7)
When at school, I worried that someone would harm me while I was changing my menstrual materials	87 (44)	81 (41)	23 (12)	8 (4)

All data obtained from both site 1 and 2

### Reuse needs and insecurity

The mean score for reuse needs (Table 6) was 2.7 (SD: 0.18); 23% had unmet reuse needs (mean score < 3). The mean score for reuse insecurity was 1.9 (SD: 0.34); 89% had unmet reuse insecurity needs. Sixty-eight (39%) reported they often or always worried that someone would see them while they were washing their menstrual materials (Table 6).

**Table 6:** Reuse needs and insecurity (n = 200)

During your last menstrual period how often did you feel that…	Participant responses, n (%)			
	Never	Sometimes	Often	Always
I had enough water to soak or wash my menstrual material	1 (1)	9 (5)	14 (8)	150 (86)
I had access to a basin to soak or wash my menstrual materials whenever I needed it	5 (3)	1 (1)	15 (9)	153 (88)
I was able to wash my menstrual materials when I wanted to	2 (1)	4 (2)	26 (15)	143 (82)
I had enough soap to wash my menstrual materials	10 (6)	22 (13)	33 (19)	109 (63)
I was able to dry my materials when I wanted to	5 (3)	4 (2)	47 (27)	118 (68)
I worried that someone would see me while I was washing my menstrual materials	37 (21)	69 (40)	41 (24)	27 (16)
I worried that my menstrual materials would not be dry when I needed them	94 (54)	51 (29)	21 (12)	8 (5)
I worried that others would see my menstrual materials while they were drying	45 (26)	76 (44)	31 (18)	22 (13)

All data obtained from both site 1 and 2

## Discussion

In this study investigating MH perspectives and practices among AYAs in rural Haiti, a majority of AYAs reported they skipped school (with any frequency) due to their menses. Further, more than half worried that something or someone would harm them while they were changing their menstrual materials at home and at school. These findings reveal potential opportunities to improve school attendance during menses, as well as access to MH materials and safe places for changing of menstrual materials for AYAs in rural Haiti.

According to the MPNS-36, more than two-thirds of participants reported unmet MH needs, with the most pressing unmet needs related to school environment (i.e. having a clean and satisfactory place to change materials), menstrual material reliability (i.e. leakage or instability of materials), and change insecurity (i.e. fear of being harmed while changing materials at home and at school). In the MPNS-36 validation study conducted among schoolgirls in Uganda, rates of unmet MH needs were similar to our findings, however, the highest proportion of unmet needs was regarding material and home environment needs (84%) [35]. In our study population, the prevalence of menstruation-related school absenteeism in rural Haiti was higher compared with similar studies among AYAs in rural settings in other LMICs (i.e., 28% [19] and 40% [20] in Ghana, and 61% [21] in Ethiopia). Some past studies assessed school absenteeism using a binary response (“yes” or “no”) while we used Likert-scale responses (never, sometimes, often, always) which may partially account for this difference [19–23]. As most schools in Haiti do not have toilets or running water, it is likely that concerns about unmet MH needs related to school environment and school attendance may be partially driven by lack of adequate WASH facilities [36]. While our study did not investigate explanatory factors, studies from other LMICs have demonstrated increased risk of MH-related absenteeism in populations with higher levels of perceived MH stigma, rural settings, lower SES, and cultural restrictions during mensturation (including exclusion from school, as well as religious events, family homes, and public sanitation facilities) [11, 16, 17, 19, 21–23, 37]. School absenteeism could also be affected by menstrual discomfort, as well as cultural shame or stigma. Thus, the high rate of school absenteeism observed in our study may be partially driven by these factors. Additionally, our study took place shortly after the earthquake that occurred in Southern Haiti on August 14, 2021, which resulted in damage or complete destruction of many schools. While some temporary learning structures (e.g., tents) have been erected, many students still do not have access to permanent school facilities. This may have impacted our study findings regarding concerns about school environment and menstruation related school absenteeism [37]. Further study into underlying drivers (e.g., menstrual discomfort, cultural stigma), as well as the impact of disasters on MH concerns about school environment and attendance is needed to inform efforts to offset MH-related school absenteeism among AYAs in Haiti. We found a high proportion of our respondents reported material reliability concerns. Among those who reponded to the questions about specific MH product use, most reported using disposable sanitary pads, or cloths/towels, which may be more prone to leakage or not staying securely in place. Future work is needed assess MH product access in Haiti and potentially develop interventions to offer higher quality MH products to young women in rural Haiti. Additionally, in our study, those who reported no religion were more likely to have a low score, indicating unmet MH needs. As numerous non-profit and humanitarian aid organizations, some of which are religiously-affiliated, offer housing, food and healthcare services in Haiti, this finding may be partially driven by women affiliated with a religion accessing resources from religiously affiliated organizations [38]. Further study is needed to better understand the association between MH resources and affiliation with a religion.

Safe spaces in home and schools for MH become more scarce in post-disaster and humanitarian settings, especially when these events lead to internally displaced people [39, 40]. Without a private and secure (e.g., lockable) space to manage menses, women are at increased risk of GBV [41–43]. More than half of our participants reported they sometimes, often, or always worried that something (e.g., animals, unsafe structures) or someone would harm them while they were changing their menstrual materials at home. As noted above, our study took place shortly after an earthquake (August 2021), which may have impacted our study participant’s reported safety concerns. Following the earthquake, approximately 13,000 houses were partially or completely destroyed [44]. Recent reports reveal that one year later, thousands of displaced families are still living in camps or under tarps and only 38 of 1250 damaged or destroyed schools have been rebuilt [45, 46]. Thus, further earthquake recovery and rebuilding needs remain which may continue to affect the security of AYAs in southern Haiti. In our study, participants who reported living in a tent were more likely to report safety concerns at home while changing their menstrual materials, compared to those living in a house. A previous study using the MPNS-36 among young Venezuelan migrant women at the Brazilian border found that 76% of women were afraid to be harmed by someone while changing their menstrual materials and 82% were afraid to be harmed by an animal or insect [47]. Displaced women are vulnerable to GBV due to increased poverty and reduced community networks [48]. A study on WASH-related needs of refugee women and girls, including Haitians, identified communal latrines, along with bathing in the bush, as dangerous, particularly at nighttime [49]. It is possible that concerns about being seen while changing or washing materials are related to the fear and threat of violence. Further study is needed to understand the underlying factors related to these MH safety concerns and risk for GBV at home and schools among young women in Haiti, particularly during and after natural disasters or political unrest.

Our findings should be viewed in light of these limitations. Data on those ineligible or who declined to participate was not collected, thus we were unable to assess for differences between those who participated and those who declined. We also added four additional questions in the midst of data collection which were only answered by participants at site 2. We enrolled a convenience sample of AYAs, thus there is risk for sampling bias and that our sample may not adequately be representative of the larger AYA population in these areas. While our sample was slightly smaller than similar studies, we did enroll across two rural geographic areas, which strengthens generalizability, particularly among other rural LMICs populations. Additionally, our study population demographics are comparable to population level data on adolescents and young adults living in Haiti, with similar rates of school attendance and educational attainment [50]. Due to the sensitivity of the topic, there is also risk for potential social desirability bias leading to inaccurate reporting. We attempted to mitigate this by conducting data collection privately and engaging trusted community members to assist with data collection. As our study included only close-ended questions, MH perspectives could be further explored using qualitative methods in the future. We used the validated MPNS-36 to assess unmet MH needs, however, it is still a relatively new MH assessment tool and normative data to date is lacking. Additionally, the MPNS-36 did not specifically assess school absenteeism, thus we added a survey question adapted from other past MH studies [35].

## Conclusions

In this study in AYA females in rural Haiti, we found that two-thirds had unmet menstrual hygiene needs, three-quarters reported they skipped school due to their menses, and more than half worried that something or someone would harm them while they were changing their menstrual materials at home and at school. While some LMICs have developed evidence-informed national policies to mobilize MH resources, our study conducted in young women living in rural Haiti highlights the need for enhanced attention to MH needs, particularly in humanitarian settings [51–54]. MPNS scoring allows us to interpret and assess the impact of specific MH met and unmet needs in our population. Our study population was affected by the earthquake (August 2021) in southern Haiti leading to loss of schools and homes, which likely may be partially responsible for certain results, including MH home and school safety concerns. Our findings may inform future humanitarian efforts during disasters in Haiti as well as other similar LMIC settings. In addition, further comprehension of underlying drivers regarding MH-related safety concerns about home and school environments as well as school attendance is needed. These findings can then inform governmental (e.g., MH policies) and non-governmental efforts (e.g., MH interventions) to improve MH in young women, particularly during disasters, to ensure access to adequate MH resources, including safe home and school environments for MH (e.g., locked latrines), and thus offset risk for adverse health and psychosocial outcomes.

## Supplementary Information

**Additional file 1.** Survey items.

**Additional file 2.** Participant demographics by MPNS-36 score.

## Data Availability

We do not plan to share individual participant data. Study protocol, analysis plan, informed consent forms and clinical study report will be made available upon request by any researchers who provide methodologically sound proposal beginning 3 months and ending 5 years following article publication.

## Abbreviations

AYA: Adolescent and young adult
MH: Menstrual health
MPNS or MPNS-36: Menstrual Practice Needs Scale
LMICs: Low- and middle-income countries
WASH: Water, sanitation, and hygiene
SES: Socio-economic status
SRH: Sexual and reproductive health
GBV: Gender-based violence
MPQ: Menstrual Practices Questionnaire
