# Supplementary material for: Menstrual health among adolescents and young adults in rural Haiti
Source: Reprod Health. 2022 Dec 20;19:227. doi: 10.1186/s12978-022-01533-4 (PMC9764460; doi:10.1186/s12978-022-01533-4)
Supplement: Supplementary file 1 — Additional file 1. Survey items. [file 12978_2022_1533_MOESM1_ESM.docx]

Appendix B: Survey Items

**Demographic Questions**

All questions are voluntary.

Are you female? Yes, continue to next

No, not eligible

How old are you in years? 14

15

16

17

18

If answers outside of age range not eligible

| **DEMOGRAPHIC INFO** |  |
| --- | --- |
| 1. **What is the highest level of school that you have finished?** | - 1. What is your highest level of education?   2. 1st grade or less   3. 1st -6th Primary school   4. 7th-9th Primary school   5. 3rd-4nd Secondary school   6. Graduated   7. I have never attended school   8. Refuse to answer |
| 1. **Do you typically go to school at least 3 days a week?** | - 1. Yes   2. No |
| 1. **What best describes where you live?** | - 1. In a house   2. Under a tent   3. Other, please specify |
| 1. **How would you best describe your religion?** | 1 Catholic  2 Other Christian religion  3 Other If other, please specify _________________  4 I do not identify with a religion. |
| **HEALTHCARE ACCESS** |  |
| 1. **Has there been any time in the past 12 months when you thought you should get medical care, but you did not?** | - 1. Yes   2. No |

**Menstrual Practice Needs Scale (MPNS)**

Hennegan J, Nansubuga A, Smith C*, et al* Measuring menstrual hygiene experience: development and validation of the Menstrual Practice Needs Scale (MPNS-36) in Soroti, Uganda

*BMJ Open*2020;**10:**e034461. doi: 10.1136/bmjopen-2019-034461

During your last menstrual period how often did you feel that?


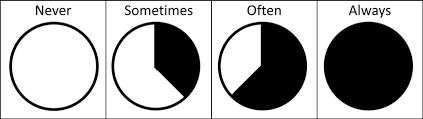


**Material and home environment needs**

2. My menstrual materials were comfortable.

6. I had enough of my menstrual materials to change them as often as I wanted to.

7. I was satisfied with the cleanliness of my menstrual materials.

8. I could get more of my menstrual materials when I needed to.

9. I felt comfortable carrying spare menstrual materials with me outside my home.

10. I felt comfortable carrying menstrual materials to the place where I changed them.

14. I felt comfortable storing my menstrual materials until my next period.

15. I was able to wash my hands when I wanted to.

23. I was able to immediately throw away my used menstrual materials or find a place to store them if they are reusable.

24. I was able to dispose of my used materials in the way that I wanted to.

25. When at home, I was able to change my menstrual materials when I wanted to.

26. When at home, I was satisfied with the place I used to change my menstrual materials.

27. When at home, I had a clean place to change my menstrual materials.

**Transport and school environment needs**

9. I felt comfortable carrying spare menstrual materials with me outside my home.

35. When at school, I was able to change my menstrual materials when I wanted to. (Include option: not currently attending school)

36. When at school, I was satisfied with the place I used to change my menstrual materials (include option: not currently attending school)

39. When at school, I had a clean place to change my menstrual materials. (Include option: not currently attending school)

40. I skipped school because I had my menses.*

*Added, not part of MPNS.

**Material reliability concerns**

3. I worried that my menstrual materials would allow blood to pass through to my outer garments.

4. I worried that my menstrual materials would move from place while I was wearing them.

5. I worried about how I would get more of my menstrual material if I ran out.

**Change and disposal insecurity**

20. I worried about where to dispose of my used menstrual materials.

22. I was concerned that others would see my used menstrual materials in the place I disposed of them.

28. When at home, I worried that I would not be able to change my menstrual materials when I needed to.

29. When at home, I worried that someone would see me while I was changing my menstrual materials.

30. When at home, I worried that someone would harm me while I was changing my menstrual materials.

31. When at home, I worried that something else would harm me while I was changing my menstrual materials (eg, animals, insects, unsafe structure).

40. When at school, I worried that I would not be able to change my menstrual materials when I needed to.

41. When at school, I worried that someone would see me while I was changing my menstrual materials.

When at school, I worried that someone would harm me while I was changing my menstrual materials.

If you washed and reused any materials during your last period, please answer these items.

43. I had enough water to soak or wash my menstrual material

I had access to a basin to soak or wash my menstrual materials whenever I needed it.

45. I was able to wash my menstrual materials when I wanted to.

I had enough soap to wash my menstrual materials.

51. I was able to dry my materials when I wanted to.

47. I worried that someone would see me while I was washing my menstrual materials.

49. I worried that my menstrual materials would not be dry when I needed them.

50. I worried that others would see my menstrual materials while they were drying.

Only answered by Site 2:

**The Menstrual Practices Questionnaire (MPQ)**

For more information see www.menstrualpracticemeasures.org and the citation below.

**Citation for the MPQ:** Hennegan, J., Nansubuga, A., Akullo, A., Smith, C., & Schwab, K.J., (2020). The Menstrual Practices Questionnaire (MPQ): Development, elaboration, and implications for future research. *Global Health Action, 13*(1), 1829402. https://doi.org/10.1080/16549716.2020.1829402

**Notes:**

1. Question wording and terms should be adapted for different languages and contexts. In particular “menstrual materials” may need to be replaced with more familiar terminology or could be replaced using computerised filters to import the menstrual material/s used.

2. All questions refer to the last menstrual period. This can be facilitated through question wording (as shown below), or the time period could be specified at the start of questioning.

3. Not all questions are applicable for all respondents, appropriate filters should be applied.

4. Question order is presented here according to topic-groupings. The order should be modified as appropriate for delivery and may be integrated with other survey questions.

**Section on menstrual material use:**

| Question | Response Options |
| --- | --- |
| What were all the materials you used to catch/absorb your menstruation when you were **at home** during your last menstrual period?  *(select all that apply)* | - Cloth/towel - Disposable sanitary pad - Reusable sanitary pad - Toilet paper - Cotton wool - Mattress or foam - Underwear alone - Natural material (e.g., leaves, sand, grass) - Period underwear - Menstrual cup - Tampon - Other (specify) |
| What were all the materials you used to catch/absorb your menstruation when you were **away from home [at school/at work]** during your last menstrual period?  *(select all that apply)* | - Cloth/towel - Disposable sanitary pad - Reusable sanitary pad - Toilet paper - Cotton wool - Mattress or foam - Underwear alone - Natural material (e.g., leaves, sand, grass) - Period underwear - Menstrual cup - Tampon - Other (specify) |
| *[Cloth users]* Were your cloths bought to be used for menstruation or used for something else first? *(select one)* | - Bought to be used during menstruation - Used for something else first (e.g., clothes, bedding) - Don’t know |
| Did you wash and reuse any of your menstrual materials during your last menstrual period? *(select one)* | • No  • Yes |
