# Supplementary material for: Menstrual health among adolescents and young adults in rural Haiti
Source: Reprod Health. 2022 Dec 20;19:227. doi: 10.1186/s12978-022-01533-4 (PMC9764460; doi:10.1186/s12978-022-01533-4)
Supplement: Supplementary file 2 — Additional file 2. Participant demographics by MPNS-36 score. [file 12978_2022_1533_MOESM2_ESM.docx]

Appendix C Participant Demographics^***^

|  | Total Scored^*^ | Low Score^**^ | High Score^**^ | p-value |
| --- | --- | --- | --- | --- |
| N=200, n (%), Responses | 174 | 26 | 148 |  |
| Age Category (years) |  |  |  | 0.56 |
| 14-17 | 48 (28) | 6 (23) | 42 (28) |  |
| 18-21 | 64 (37) | 12 (46) | 52 (35) |  |
| 22-24 | 62 (36) | 8 (31) | 54 (36) |  |
| Highest education level (grade) |  |  |  | 0.35 |
| 1-6th primary school | 32 (18) | 6 (23) | 26 (18) |  |
| 7-9th primary school | 66 (38) | 6 (23) | 60 (41) |  |
| 3-4th secondary school | 67 (39) | 13 (50) | 54 (36) |  |
| Graduated secondary school | 9 (5) | 1 (4) | 8 (5) |  |
| Marital status |  |  |  | 1.00 |
| Married | 7 (4) | 1 (4) | 6 (4) |  |
| Regular school attendance (attending school at least 3 days a week) |  |  |  | 0.40 |
| Yes | 87 (50) | 11 (42) | 76 (51) |  |
| Living situation |  |  |  | 0.75 |
| House | 152 (87) | 22 (85) | 130 (88) |  |
| Tent | 22 (13) | 4 (15) | 18 (12) |  |
| Religion |  |  |  | <0.01 |
| Catholic | 59 (34) | 4 (15) | 55 (37) |  |
| Other Christian religion | 82 (47) | 10 (38) | 72 (49) |  |
| Other | 0 (0) | 0 (0) | 0 (0) |  |
| None | 32 (18) | 11 (42) | 21 (14) |  |
| Did not answer | 1 (1) | 1 (4) | 0 (0) |  |
| Sexual Orientation |  |  |  | 1.00 |
| Gay | 0 (0) | 0 (0) | 0 (0) |  |
| Lesbian | 0 (0) | 0 (0) | 0 (0) |  |
| Straight/not gay or lesbian | 174 (100) | 26 (100) | 148 (100) |  |
| Bisexual | 0 (0) | 0 (0) | 0 (0) |  |
| Did not seek care in past year when felt they should | 89 (51) | 18 (69) | 71 (48) | 0.13 |

^*^Responses with 3 or more missing items were not scored

^**^Low scores were defined as a total mean score <1.82; high scores were defined as a total mean score > 1.82

^***^All data obtained from both site 1 and 2
